# Antifungal Activity and Mode of Action of Miltefosine Against Clinical Isolates of *Candida krusei*

**DOI:** 10.3389/fmicb.2020.00854

**Published:** 2020-05-19

**Authors:** Yongqin Wu, Mengying Wu, Jing Gao, Chunmei Ying

**Affiliations:** Department of Clinical Laboratory, Obstetrics and Gynecology Hospital of Fudan University, Shanghai, China

**Keywords:** *Candida krusei*, susceptibility, miltefosine, synergy, mode of action

## Abstract

*Candida krusei* attracts attention from medical professionals mainly for its intrinsic resistance to fluconazole and the limited number of drugs available to treat *C. krusei* vulvovaginal candidiasis. Miltefosine was demonstrated to have good antifungal activity both *in vitro* and *in vivo*. Here, we determined the susceptibility profiles of 57 clinical *C. krusei* isolates from vulvovaginal candidiasis patients and assessed the antifungal activity of miltefosine against *C. krusei*. All isolates were susceptible to voriconazole and itraconazole, whereas 1.8% of the isolates were of non-wild-type phenotype to amphotericin B. In contrast, miltefosine showed low MICs against all *C. krusei* isolates with fungicidal activity. The checkerboard assay showed that the synergistic effect of miltefosine in combination with amphotericin B was observed in 25% of the tested planktonic *C. krusei* isolates and 18.8% of the tested preformed biofilms, whereas miltefosine in combination with fluconazole showed indifferent interaction for all tested planktonic isolates. The presence of sorbitol in the broth microdilution assay did not influence the MIC values of miltefosine against *C. krusei*, but the presence of ergosterol increased the MIC values. Visible changes in cell content in cells treated with miltefosine were observed. We found that cells treated with miltefosine showed decreased cell viability and chromatin condensation under PI staining, which indicates that miltefosine may induce apoptosis-like cell death in *C. krusei*. In conclusion, we found miltefosine has a good activity against *C. krusei* isolates and exerts its fungicidal effect by binding to ergosterol in the cell membrane and inducing apoptosis.

## Introduction

Pathogenic *Candida* species can cause invasive candidiasis that takes more than 50,000 lives worldwide annually, and it also causes recurrent vulvovaginal candidiasis that affects approximately 138 million women annually (Kullberg and Arendrup, 2015; Denning et al., 2018). *Candida albicans* candidiasis infections are the most well-known, but non-*albicans* candidiasis infections have also increased in recent years (Lamoth et al., 2018). *Candida krusei* candidiasis infections are of particular concern because *C. krusei* is inherently resistant to fluconazole, a common drug used for antifungal treatment, and *C. krusei* fungemia can cause high mortality rates ranging from 60 to 80% (Abbas et al., 2000; Lamping et al., 2009; Schuster et al., 2013). Finding an effective way of controlling vulvovaginal candidiasis caused by fluconazole-resistant *Candida* species is an important problem because there are only a few effective therapeutic drugs that can be used on vulvovaginal candidiasis patients (Sobel, 2016; Sobel and Sobel, 2018).

Although echinocandins such as caspofungin are currently the drugs of choice for treatment of invasive *C. krusei* candidiasis infections, echinocandin-resistant isolates caused by point mutations in the *FKS1* gene have increased (Jensen et al., 2014; Forastiero et al., 2015; Lallitto et al., 2018). Some studies have shown that *C. krusei* has considerable resistance to the antifungal drugs itraconazole and amphotericin B, and others have shown that it has become a multidrug-resistant pathogen (Pfaller et al., 2008; He et al., 2015; Ricardo et al., 2020). It has become essential to develop antifungal agents with novel antifungal mechanisms for the treatment of candidiasis caused by *C. krusei*.

Miltefosine, an alkylphosphocholine, was initially developed as an antitumor drug, but it is now a clinically licensed antiparasitic drug (Dorlo et al., 2012). Miltefosine is an oral anti-*Leishmania* drug, and the pharmacokinetics of miltefosine in children and adults showed high miltefosine plasma concentrations (i.e., 17–42 μg/mL) for the treatment of cutaneous leishmaniasis (Castro et al., 2017). Miltefosine is a less toxic drug; some clinical trials have shown that no treatment-related serious adverse events were reported for the treatment of leishmaniasis both in children and adults, although adverse events were usually reported (Wasunna et al., 2016; Ventin et al., 2018; Mbui et al., 2019).

Miltefosine has also been found to possess antifungal potential; both *in vitro* and *in vivo* activities against *Cryptococcus* yeasts have been observed (Widmer et al., 2006). The *in vitro* antifungal activities of miltefosine against dermatophytes, *Paracoccidioides* yeasts, and *Candida auris* were found one after another (Tong et al., 2007; Rossi et al., 2017; Wu et al., 2020). It has been reported that miltefosine has the *in vivo* efficacy in a murine model of oral candidiasis caused by *C. albicans* (Vila et al., 2015). In previous studies, miltefosine displayed good therapeutic effects against vaginal candidiasis in mice and against candidiasis and cryptococcosis in the larval models of *Galleria mellonella* (de Bastiani et al., 2019; Spadari et al., 2019). Many studies have established the activity of miltefosine against *Candida* species planktonic cells and biofilms (Vila et al., 2013, 2016), but the antifungal effect of miltefosine against *C. krusei* has not been reported.

Data on susceptibility profiles of *C. krusei* isolates from patients with vulvovaginal candidiasis are limited. In this work, we first assessed the antifungal susceptibility patterns of *C. krusei* isolates from vulvovaginal candidiasis patients. We examined the antifungal activity of miltefosine against 57 clinical isolates of *C. krusei*. We assessed the efficacy of miltefosine in combination with azoles and amphotericin B against *C. krusei* planktonic cells and biofilms. We also evaluated the potential mechanism of action of miltefosine against *C. krusei*.

## Materials and Methods

### Strains, Media, and Growth Conditions

Clinical *C. krusei* isolates were collected from vulvovaginal candidiasis patients as part of routine screening, and ethical approval for their use was not required as per local/national guidelines, and those isolates were stocked in our laboratory. Patient consent was not required as samples were obtained as part of routine screening. All isolates were stored with 15% glycerol at −80°C. *Candida krusei* strains routinely grew on YPD (1% yeast extract, 2% peptone, 2% glucose) media at 30°C. All of the isolates were identified using matrix-assisted laser desorption/ionization time-of-flight mass spectrometry (Bruker, Karlsruhe, Germany). RPMI 1640 broth was made for drug susceptibility testing. Miltefosine (Sigma-Aldrich, San Francisco, CA, United States) was dissolved in distilled water and stored at room temperature. The ATCC 6258 strain and ATCC 22019 strains were selected as controls for antifungal susceptibility testing.

### Antifungal Susceptibility Testing

The drug minimum inhibitory concentrations (MICs) of the 57 clinical isolates were determined using the broth microdilution method established by the Clinical & Laboratory Standards Institute (CLSI) M27-A4 document (2017). Minimum inhibitory concentration results for voriconazole were interpreted using clinical breakpoints according to CLSI M60 document (CLSI, 2017), and those for amphotericin B and itraconazole were interpreted using epidemiological cutoff values according to CLSI M59 2nd document (CLSI, 2018). The MIC was defined as the lowest concentration producing 100% growth inhibition for amphotericin B and miltefosine. The endpoint was defined as at least 50% inhibition of growth for voriconazole, itraconazole, and fluconazole. All isolates were incubated at 35°C, and results were read at 24 h for all drugs. For the minimum fungicidal concentrations, after MICs were read, 100 μL of suspension of each well with drug concentrations above the MIC was then plated on YPD plates for 2 days of incubation. Minimum fungicidal concentration was defined as the lowest drug concentration that eliminated 99.9% of the colonies formed on the plates. Quality control was performed for each drug set every time. We repeated all testing three times, and when any two results were consistent, we reported the consistent result.

### Antifungal Synergism Testing

Testing for antifungal drug interactions was performed in accordance with the broth microdilution checkerboard method based on aforementioned CLSI M27 document. The final concentration of antifungal drugs ranged from 0.015 to 16 μg/mL for amphotericin B and miltefosine and 1–128 μg/mL for fluconazole. Minimum inhibitory concentration readings were performed at 24 h of incubation. The 100% inhibition endpoint was used for the combination of amphotericin B and miltefosine. And the 50% or more inhibition endpoint was used for miltefosine and fluconazole combinations. To assess the *in vitro* interactions between antifungal drugs, the fractional inhibitory concentration index (FICI) was calculated. The interactions were defined as synergistic if FICI ≤0.5, indifferent if 0.5 < FICI < 4.0, and antagonistic if FICI > 4.0 (Odds, 2003).

### Susceptibility Testing of Preformed Biofilms

Preformed biofilms were grown as described previously with some modifications (Pierce et al., 2008). Logarithmic growth phase of *C. krusei* cells was harvested and washed three times with phosphate-buffered saline (PBS). Pellets were then resuspended in RPMI-1640 medium with the final density of 1 × 10^6^ cells/mL. A total of 100 μL of the *C. krusei* suspension was pipetted into 96-well clear flat-bottom polystyrene TC-treated microplates (Corning, NY, United States) and incubated at 37°C for 24 h. After incubation, preformed biofilms were washed three times with PBS, and then drugs were added to the wells at different concentrations. The plates were incubated for another 24 h, and the MICs of preformed biofilm were defined as the lowest concentration producing at least 80% reduction in metabolic activity of cells using XTT [2,3-*bis*-(2-methoxy-4-nitro-5-sulfophenyl)-2*H*-tetrazolium-5-carboxanilide] reduction assay as described previously (Kovacs et al., 2016).

### Sorbitol Assay and Ergosterol Assay

To assess the effect of miltefosine on the cell wall or cell membrane of *C. krusei*, the sorbitol assay and ergosterol assay were performed as described previously (Turecka et al., 2018). The MICs were assessed in the presence of 0.8 M sorbitol or 200 μg/mL ergosterol according to the CLSI document named above. Each well supplemented with sorbitol was incubated at 35°C for 2 and 7 days. And wells supplemented with ergosterol were incubated at 35°C for 24 h. Caspofungi against *C. krusei* ATCC 6258 were used as a positive control in sorbitol assay, and amphotericin B was used as a positive control in ergosterol assay.

### Light Microscopy Analysis of Cell Morphology

Cell suspensions at 1 × 10^6^ cells/mL in YPD were treated with 0, 2, and 4 μg/mL miltefosine for 12 h. Then cells were washed and then analyzed using light microscopy.

### Cell Chromatin Condensation and Cell Viability Analyses

Briefly, the cells [1 × 10^7^ colony-forming units (CFU)/mL] were treated with 0, 2, and 4 μg/mL miltefosine in PBS for 5 h. The nuclear condensation was analyzed using propidium iodide (PI) staining. After treatment, cells were washed, suspended in PBS, and incubated with 2 μg/mL PI at the room temperature in the dark for 10 min. Then cells were determined using a fluorescence microscope (Olympus, Tokyo, Japan). To determine cell viability, the cells after treatment were washed, diluted serially, and plated on YPD plates. The plates were incubated for 2 days, and then percentage of survival was evaluated based on CFU counting.

### Statistical Analysis

Statistical analyses were performed using Student *t* tests by GraphPad Prism 8.0 (GraphPad Software, CA, USA). Differences were considered significant at *P* < 0.05.

## Results

### Antifungal Susceptibility of Clinical *C. krusei* Isolates

The details of comparative susceptibilities to the four common antifungals and miltefosine against 57 *C. krusei* isolates from vulvovaginal candidiasis patients are shown in Supplementary Table S1. There was no obvious difference in the susceptibility patterns of *C. krusei* isolates from different years. The antifungal descriptive statistic values for *C. krusei* isolates are shown in Table 1. All isolates were inherently resistant to fluconazole (MICs ≥ 16 μg/mL; Table 1). All isolates were susceptible and of wild type to voriconazole and itraconazole, respectively. The MIC_90_ values for voriconazole and itraconazole were 0.25 and 0.5 μg/mL, respectively, and the geometric mean was 0.119 and 0.351 μg/mL, respectively. However, amphotericin B did not show good activity against *C. krusei* isolates, with MIC_90_ values of 2 μg/mL and geometric means of 1.905 μg/mL. One isolate (1.8%) was interpreted as non-wild-type phenotype (MIC = 4 μg/mL) to amphotericin B. Miltefosine was found to have activity against clinical *C. krusei* isolates with MIC_90_ value of 2 μg/mL. Its geometric mean value and MIC range were 1.882 and 0.5–2 μg/mL, respectively. Miltefosine showed fungicidal activity against *C. krusei* isolates (Supplementary Table S1).

**TABLE 1:** Antifungal descriptive statistic values for 57 clinical *C. krusei* isolates.

Drug	Range (μ g/mL)	Median	Mode	MIC_50_	MIC_90_	GM (μ g/mL)
ITR	0.125–1	0.5	0.5	0.5	0.5	0.351
VOR	0.063–0.5	0.125	0.125	0.125	0.25	0.119
FLC	16–128	32	32	32	64	39.830
AMB	1–4	2	2	2	2	1.905
MIL	0.5–2	2	2	2	2	1.882

*Median, the value in the middle of the rank; Mode, the value that occurs at the greatest frequency; MIC_50_ or MIC_90_, minimal inhibitory concentration of 50% or 90%; GM, geometric mean; ITR, itraconazole; VOR, voriconazole; FLC, fluconazole; AMB, amphotericin B; MIL, miltefosine.*

### Antifungal Synergy Testing for *C. krusei* Planktonic Cells

We randomly chose 20 isolates for synergy testing of miltefosine and common antifungal drugs against planktonic *C. krusei* isolates by the checkerboard method. Miltefosine and fluconazole combinations showed no synergistic effects against *C. krusei* isolates (Table 2). When miltefosine was tested with amphotericin B, synergistic effects were observed in five isolates (25% of the isolates) but not including the amphotericin B non-wild-type isolate (Table 2). However, when miltefosine and amphotericin B combination was tested, the MIC of amphotericin B decreased at least eightfold (from 2 to 0.25 μg/mL) in 16 isolates (80% of the isolates).

**TABLE 2:** The synergy testing results of antifungal drugs against *C. krusei* planktonic cells based on the fractional inhibitory concentration index (FICI) values.

(a)									
	MIC (μ g/mL) of					MIC (μ g/mL) of			
ID	AMB	MIL	AMB/MIL	FICI^*a*^	ID	AMB	MIL	AMB/MIL	FICI^*a*^
3	2	2	0.25/1	0.63	37	2	2	0.25/1	0.63
11	2	2	0.5/0.5	**0.5**	39	2	2	0.25/1	0.63
17	2	1	0.25/1	1.13	41	2	2	0.5/0.5	**0.5**
21	2	2	0.25/1	0.63	42	4	2	0.5/1	0.63
24	2	2	0.25/1	0.63	45	2	2	0.125/0.5	**0.38**
25	2	2	0.25/1	0.63	47	2	2	0.25/1	0.63
27	2	2	0.25/1	0.63	49	2	2	0.5/0.5	**0.5**
30	1	2	0.125/0.5	**0.38**	51	2	2	0.25/1	0.63
32	2	2	0.25/1	0.63	54	2	2	0.25/1	0.63
34	2	2	0.25/1	0.63	57	2	2	0.25/1	0.63

**(b)**									
	**MIC (μ g/mL) of**					**MIC (μ g/mL) of**			
**ID**	**FLC**	**MIL**	**FLC/MIL**	**FICI^*a*^**	**ID**	**FLC**	**MIL**	**FLC/MIL**	**FICI^*a*^**

3	32	2	>16/>1	2	37	128	2	>64/>1	2
11	16	2	>8/>1	2	39	64	2	>32/>1	2
17	32	1	>16/>0.5	2	41	64	2	>32/>1	2
21	32	2	>16/>1	2	42	128	2	>64/>1	2
24	64	2	>32/>1	2	45	64	2	>32/>1	2
25	32	2	>16/>1	2	47	64	2	>32/>1	2
27	32	2	>16/>1	2	49	32	2	>16/>1	2
30	64	2	>32/>1	2	51	32	2	>16/>1	2
32	32	2	>16/>1	2	54	32	2	>16/>1	2
34	32	2	>16/>1	2	57	64	2	>32/>1	2

*^*a*^FICI values in bold indicate synergy. AMB, amphotericin B; FLC, fluconazole; MIL, miltefosine.*

### *In vitro* Interactions of Miltefosine in Combination With Amphotericin B Against *C. krusei* Preformed Biofilms

Treatment for preformed biofilms is difficult for hospitalized patients. For this reason, we examined the synergistic activity of miltefosine in combination with amphotericin B on preformed (24-h-old) biofilms of 16 randomly selected *C. krusei* isolates. Synergy of miltefosine in combination with amphotericin B was observed for three isolates (18.8% of the isolates) when grown in biofilm (Table 3). Although indifferent effects were observed in the rest of these isolates, the MIC of amphotericin B was reduced eightfold (from 16 to 2 μg/mL) for isolates 25 and 37 and fourfold for other isolates (Table 3).

**TABLE 3:** *In vitro* synergy testing results of miltefosine in combination with amphotericin B against *C. krusei* preformed biofilms.

	MIC (μ g/mL) of					MIC (μ g/mL) of			
ID	AMB	MIL	AMB/MIL^*a*^	FICI^*b*^	ID	AMB	MIL	AMB/MIL^*a*^	FICI^*b*^
3	8	8	2/4	0.75	37	16	8	2/2	**0.375**
11	8	8	2/2	**0.5**	39	16	16	8/4	0.75
17	16	8	8/2	0.75	41	16	8	4/4	0.75
21	16	16	8/4	0.75	42	8	8	4 / 2	0.75
24	8	8	4/2	0.75	45	16	8	4/2	**0.5**
25	16	8	2/4	0.625	47	16	8	2/4	0.625
27	8	8	2/4	0.75	49	8	8	2/4	0.75
30	8	8	2/4	0.75	51	16	8	4/4	0.75

*^*a*^The 80% inhibition endpoint was used for this combination. ^*b*^FICI values in bold indicate synergy. AMB, amphotericin B; MIL, miltefosine; FICI, fractional inhibitory concentration index.*

### Exogenous Ergosterol Instead of Sorbitol Reduces the Antifungal Effect of Miltefosine on *C. krusei* Strains

In order to explore the antifungal mode of action of new drug, exogenous ergosterol and the osmotic protection against the antifungal effect were analyzed as described previously (Pereira et al., 2016; Turecka et al., 2018). We next determined whether the addition of exogenous sorbitol (an osmotic protector) or ergosterol to the culture medium would increase the MIC values of miltefosine against *C. krusei* isolates. The presence of sorbitol in the broth microdilution assay did not alter the MIC values of miltefosine against tested clinical isolates in the 2- or 7-day group, as did caspofungin (Table 4). However, an eightfold or fourfold MIC increase in miltefosine was observed after the addition of ergosterol in broth microdilution assay among tested isolates (Table 4). This behavior is similar to the case of amphotericin B (positive control), which binds directly to ergosterol on the fungal membrane. In this way, miltefosine may bind to ergosterol in the fungal cell membrane instead of in the cell wall biosynthesis pathway.

**TABLE 4:** Effect of exogenous sorbitol and ergosterol on the MIC (μg/mL) values of miltefosine against clinical *C. krusei* isolates.

Isolates	Sorbitol			Ergosterol	
ID	Absence	Presence		Absence	Presence
		2 days	7 days		
3	2	2	2	2	16
17	1	1	1	1	8
25	2	2	2	2	8
36	2	2	2	2	16
43	2	2	2	2	8
52	2	2	2	2	8
ATCC6258	2	2	2	2	16
*CAS	0.25	>8	>8	–	–
^#^AMB	–	–	–	1	>16

*CAS, caspofungin, positive control for sorbitol assay; ^#^AMB, amphotericin B, positive control for ergosterol assay.

### Miltefosine Treatment Alters the Cell Content and Induces Apoptosis-Like Cell Death in *C. krusei*

**FIGURE 1:**
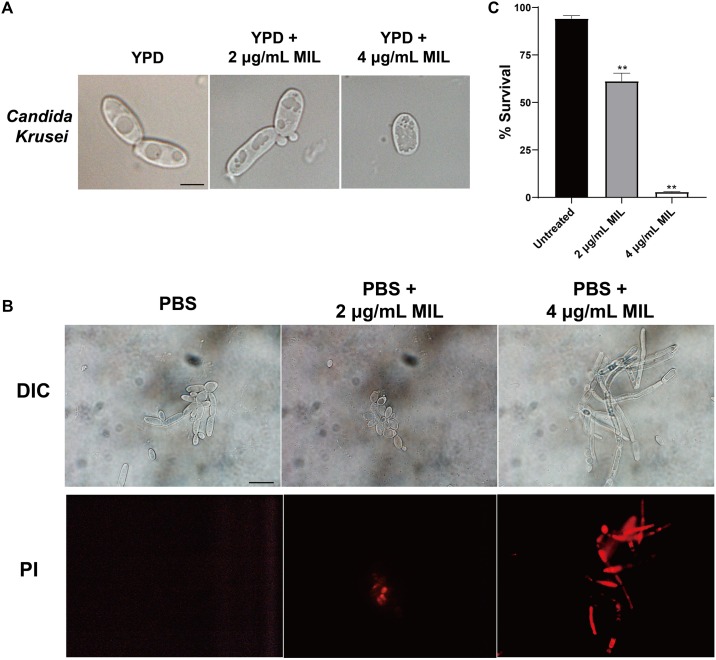
Miltefosine treatment alters the cell content and induces apoptosis-like cell death in *C. krusei*. **(A)** The *C. krusei* cells of a representative isolate with or without miltefosine treatment were analyzed by light microscopy. Scale bars = 10 μm. **(B)** Cell chromatin condensation was observed by PI staining. Scale bars = 20 μm. **(C)** Percentage of surviving cells was evaluated by CFU counting after treatment with miltefosine. **P < 0.01 compared with untreated cells.

The effect of miltefosine on the cell morphology of *C. krusei* strains was assessed using light microscopy. Unlike the untreated cells, the yeast cells treated with miltefosine showed visible changes in cell content (Figure 1A). We also tested the yeast-to-hypha transition of *C. krusei* strains using Spider medium and RPMI 1640 medium, but we did not observe the morphological changes of planktonic cells in hypha-inducing media (data not shown). We also assessed cell apoptosis after miltefosine treatment using PI staining. It was found that cells treated with miltefosine showed red fluorescent spots that indicate chromatin condensation, whereas the untreated cells did not display any fluorescence (Figure 1B). And the cells after treatment exhibited significantly lower cell survival rates compared to the untreated group (Figure 1C), which indicates miltefosine could induce *C. krusei* cell death quickly.

## Discussion

In this study, we assessed the susceptibility of azoles and amphotericin B against *C. krusei* isolates from patients with vulvovaginal candidiasis. We found all isolates were susceptible and of wild type to voriconazole and itraconazole, respectively, which is consistent with other reports on the susceptibility of isolates from invasive candidiasis patients (Gong et al., 2018). However, 1.8% of these isolates were of non-wild-type phenotype to amphotericin B. Most of them had a low susceptibility to amphotericin B and had a narrower MIC range than has been reported for isolates from invasive candidiasis patients (Gong et al., 2018; Israel et al., 2019).

In contrast, miltefosine showed low MICs against all *C. krusei* isolates and had fungicidal activity. The MIC_90_ of our collection of 57 isolates was 2 μg/mL, and its geometric mean was only 1.882 μg/mL. According to the data on clinical safety and pharmacokinetics of miltefosine (Castro et al., 2017; Mbui et al., 2019), the MIC value of miltefosine in this study is much lower than the plasma concentration observed during the treatment of leishmaniasis patients. In addition, miltefosine was shown to have significant antifungal activity against *C. albicans in vitro* and *in vivo* (Vila et al., 2015; de Bastiani et al., 2019; Spadari et al., 2019). This indicates that miltefosine should be a good candidate for *in vivo* studies in the future.

We also tested the synergistic activity of miltefosine in combination with azoles and amphotericin B against *C. krusei* isolates. No synergistic effects were found in the group of miltefosine and azoles (data not shown). In contrast, miltefosine has been reported to have synergy with azoles against *Fusarium oxysporum* and the mucormycetes (Biswas et al., 2013). Here, 25% of the tested isolates were observed to have synergistic activity of miltefosine in combination with amphotericin B against planktonic *C. krusei* isolates. These synergistic effects were consistent with our previous report in *Candida auris* (Wu et al., 2020).

Biofilm drug resistance in vulvovaginal candidiasis biofilms is an important virulence factor and a thorny issue for the treatment of vulvovaginal candidiasis (Rodriguez-Cerdeira et al., 2019). It has been reported that miltefosine is effective against biofilms of four *Candida* species (Vila et al., 2016). We found 18.8% of the tested isolates were observed to have synergistic activity of miltefosine in combination with amphotericin B against *C. krusei* preformed biofilms in this study. We also found miltefosine increased the antifungal activity of amphotericin B against most clinical isolates, both planktonic cells and preformed biofilms, by eightfold. Miltefosine in combination with amphotericin B may be a good choice for the treatment of biofilm-related candidiasis, but *in vivo* studies need to be used to evaluate the treatment efficacy in the future.

It has been already established that miltefosine interacts with cell membrane lipids such as sterols in *Leishmania* cells and cancer cells (Barratt et al., 2009). To determine whether ergosterol of the fungal cell membrane is the target of miltefosine, we performed an ergosterol competition assay. We found that exogenous ergosterol in the culture medium produced MIC values of miltefosine against *C. krusei* cells higher than those produced in ergosterol-free medium, which is consistent with previous studies performed on *Cryptococcus* yeasts (Spadari et al., 2018). The presence of sorbitol in the medium can provide a protective environment for the cell wall biosynthesis pathway by maintaining the proper osmotic pressure (Turecka et al., 2018). But no differences in MIC values were observed in either the presence or absence of sorbitol. This suggests miltefosine may not be involved in the cell wall biosynthesis pathway. Those results indicate that the ergosterol of fungal cell membrane may be part of the mode of action of miltefosine against *C. krusei* cells.

In addition, it has been reported that miltefosine has fungicidal activity by inducing apoptosis in *Cryptococcus* species and yeast (Zuo et al., 2011; Spadari et al., 2018). Nuclear condensation is one of the hallmarks of apoptosis. We examined nuclear condensation and survival rates of *C. krusei* cells with and without miltefosine treatment, and we observed more nuclear condensation and less cell viability in *C. krusei* cells treated with miltefosine. These results showed that miltefosine could induce apoptosis-like cell death in *C. krusei*. Therefore, apoptosis may be an important mechanism by which miltefosine exerts its antifungal activity against *C. krusei*. However, further studies are required to explore the exact mechanism of action of miltefosine against *Candida* species.

In conclusion, this study demonstrated that miltefosine has a good activity as a fungicidal agent against *C. krusei* isolates. Synergistic effects against both planktonic *C. krusei* cells and biofilms were observed in some isolates when miltefosine was combined with amphotericin B. Mechanism experiments showed that miltefosine may bind to ergosterol in the fungal cell membrane and induce apoptosis-like cell death in *C. krusei*. These findings may provide insight into the possible therapeutic application of miltefosine, but further *in vivo* studies need to be performed.

## Data Availability Statement

All datasets generated for this study are included in the article/Supplementary Material.

## Author Contributions

YW, MW, and JG performed the experiments. YW, MW, and CY analyzed the data. YW and CY wrote the manuscript with support from all authors.

## Conflict of Interest

The authors declare that the research was conducted in the absence of any commercial or financial relationships that could be construed as a potential conflict of interest.

## References

[B1] Abbas J. Bodey G. P. Hanna H. A. Mardani M. Girgawy E. Abi-Said D. (2000). *Candida krusei* fungemia. An escalating serious infection in immunocompromised patients. *Arch. Intern. Med.* 160 2659–2664. 10.1001/archinte.160.17.2659 10999981

[B2] Barratt G. Saint-Pierre-Chazalet M. Loiseau P. M. (2009). Cellular transport and lipid interactions of miltefosine. *Curr. Drug Metab.* 10 247–255. 10.2174/138920009787846332 19442087

[B3] Biswas C. Sorrell T. C. Djordjevic J. T. Zuo X. Jolliffe K. A. Chen S. C. (2013). In vitro activity of miltefosine as a single agent and in combination with voriconazole or posaconazole against uncommon filamentous fungal pathogens. *J. Antimicrob. Chemother.* 68 2842–2846. 10.1093/jac/dkt282 23861311

[B4] Castro M. D. Gomez M. A. Kip A. E. Cossio A. Ortiz E. Navas A. (2017). Pharmacokinetics of miltefosine in children and adults with cutaneous leishmaniasis. *Antimicrob. Agents Chemother.* 61:16. 10.1128/AAC.02198-16 27956421 PMC5328512

[B5] CLSI (2017). *Performance Standards for Antifungal Susceptibility Testing of Yeasts. CLSI Supplement M*60. Wayne, PA: Clinical and Laboratory Standards Institute.

[B6] CLSI (2018). *Epidemiological Cutoff Values for Antifungal Susceptibility Testing. CLSI Supplement M59*, 2nd Edn, Wayne, PA: Clinical and Laboratory Standards Institute.

[B7] de Bastiani F. Spadari C. C. de Matos J. Salata G. C. Lopes L. B. Ishida K. (2019). Nanocarriers provide sustained antifungal activity for amphotericin B and miltefosine in the topical treatment of murine vaginal candidiasis. *Front. Microbiol.* 10:2976 10.3389/fmicb.2019.02976 PMC6965356 31998264

[B8] Denning D. W. Kneale M. Sobel J. D. Rautemaa-Richardson R. (2018). Global burden of recurrent vulvovaginal candidiasis: a systematic review. *Lancet Infect. Dis.* 18 e339–e347. 10.1016/S1473-3099(18)30103-8 30078662

[B9] Dorlo T. P. Balasegaram M. Beijnen J. H. de Vries P. J. (2012). Miltefosine: a review of its pharmacology and therapeutic efficacy in the treatment of leishmaniasis. *J. Antimicrob. Chemother.* 67 2576–2597. 10.1093/jac/dks275 22833634

[B10] Forastiero A. Garcia-Gil V. Rivero-Menendez O. Garcia-Rubio R. Monteiro M. C. Alastruey-Izquierdo A. (2015). Rapid development of *Candida krusei* echinocandin resistance during caspofungin therapy. *Antimicrob. Agents Chemother.* 59 6975–6982. 10.1128/AAC.01005-15 26324281 PMC4604417

[B11] Gong J. Xiao M. Wang H. Kudinha T. Wang Y. Zhao F. (2018). Genetic differentiation, diversity, and drug susceptibility of *Candida krusei*. *Front. Microbiol.* 9:2717 10.3389/fmicb.2018.02717 PMC6256198 30524386

[B12] He X. Zhao M. Chen J. Wu R. Zhang J. Cui R. (2015). Overexpression of both ERG11 and ABC2 genes might be responsible for itraconazole resistance in clinical isolates of *Candida krusei*. *PLoS One* 10:e0136185. 10.1371/journal.pone.0136185 26308936 PMC4550294

[B13] Israel S. Amit S. Israel A. Livneh A. Nir-Paz R. Korem M. (2019). The epidemiology and susceptibility of candidemia in jerusalem, Israel. *Front. Cell Infect. Microbiol.* 9:352. 10.3389/fcimb.2019.00352 31681629 PMC6801307

[B14] Jensen R. H. Justesen U. S. Rewes A. Perlin D. S. Arendrup M. C. (2014). Echinocandin failure case due to a previously unreported FKS1 mutation in *Candida krusei*. *Antimicrob. Agents Chemother.* 58 3550–3552. 10.1128/AAC.02367-14 24687511 PMC4068455

[B15] Kovacs R. Bozo A. Gesztelyi R. Doman M. Kardos G. Nagy F. (2016). Effect of caspofungin and micafungin in combination with farnesol against Candida parapsilosis biofilms. *Int. J. Antimicrob. Agents* 47 304–310. 10.1016/j.ijantimicag.2016.01.007 26968084

[B16] Kullberg B. J. Arendrup M. C. (2015). Invasive candidiasis. *N. Engl. J. Med.* 373 1445–1456. 10.1056/NEJMra1315399 26444731

[B17] Lallitto F. Prigitano A. Mangione F. Piralla A. Tamarozzi F. Marone P. (2018). Presence of L701M mutation in the FKS1 gene of echinocandin-susceptible *Candida krusei* isolates. *Diagn. Microbiol. Infect. Dis.* 92 311–314. 10.1016/j.diagmicrobio.2018.07.005 30131237

[B18] Lamoth F. Lockhart S. R. Berkow E. L. Calandra T. (2018). Changes in the epidemiological landscape of invasive candidiasis. *J. Antimicrob. Chemother.* 73 i4–i13. 10.1093/jac/dkx444 29304207 PMC11931512

[B19] Lamping E. Ranchod A. Nakamura K. Tyndall J. D. Niimi K. Holmes A. R. (2009). Abc1p is a multidrug efflux transporter that tips the balance in favor of innate azole resistance in *Candida krusei*. *Antimicrob. Agents Chemother.* 53 354–369. 10.1128/AAC.01095-08 19015352 PMC2630665

[B20] Mbui J. Olobo J. Omollo R. Solomos A. Kip A. E. Kirigi G. (2019). Pharmacokinetics, safety, and efficacy of an allometric miltefosine regimen for the treatment of visceral leishmaniasis in eastern african children: an open-label, Phase II clinical trial. *Clin. Infect. Dis.* 68 1530–1538. 10.1093/cid/ciy747 30188978 PMC6481997

[B21] Odds F. C. (2003). Synergy, antagonism, and what the chequerboard puts between them. *J. Antimicrob. Chemother.* 52:1. 10.1093/jac/dkg301 12805255

[B22] Pereira J. V. Freires I. A. Castilho A. R. Da C. M. Alves H. S. Rosalen P. L. (2016). Antifungal potential of *Sideroxylon obtusifolium* and *Syzygium cumini* and their mode of action against *Candida albicans*. *Pharm. Biol.* 54 2312–2319. 10.3109/13880209.2016.1155629 26987037

[B23] Pfaller M. A. Diekema D. J. Gibbs D. L. Newell V. A. Nagy E. Dobiasova S. (2008). *Candida krusei*, a multidrug-resistant opportunistic fungal pathogen: geographic and temporal trends from the ARTEMIS DISK antifungal surveillance program, 2001 to 2005. *J. Clin. Microbiol.* 46 515–521. 10.1128/JCM.01915-07 18077633 PMC2238087

[B24] Pierce C. G. Uppuluri P. Tristan A. R. Wormley F. J. Mowat E. Ramage G. (2008). A simple and reproducible 96-well plate-based method for the formation of fungal biofilms and its application to antifungal susceptibility testing. *Nat. Protoc.* 3 1494–1500. 10.1038/nport.2008.141 18772877 PMC2741160

[B25] Ricardo E. Grenouillet F. Miranda I. M. Silva R. M. Eglin G. Devillard N. (2020). Mechanisms of acquired in vivo and in vitro resistance to voriconazole by *Candida krusei* following exposure to suboptimal drug concentration. *Antimicrob. Agents Chemother.* 64:e01651-19. 10.1128/AAC.01651-19 31932372 PMC7179295

[B26] Rodriguez-Cerdeira C. Gregorio M. C. Molares-Vila A. Lopez-Barcenas A. Fabbrocini G. Bardhi B. (2019). Biofilms and vulvovaginal candidiasis. *Coll. Surf. B Biointerf.* 174 110–125. 10.1016/j.colsurfb.2018.11.011 30447520

[B27] Rossi D. Spadari C. C. Nosanchuk J. D. Taborda C. P. Ishida K. (2017). Miltefosine is fungicidal to *Paracoccidioides* spp. yeast cells but subinhibitory concentrations induce melanisation. *Int. J. Antimicrob. Agents* 49 465–471. 10.1016/j.ijantimicag.2016.12.020 28279786

[B28] Schuster M. G. Meibohm A. Lloyd L. Strom B. (2013). Risk factors and outcomes of *Candida krusei* bloodstream infection: a matched, case-control study. *J. Infect.* 66 278–284. 10.1016/j.jinf.2012.11.002 23174708

[B29] Sobel J. D. (2016). Recurrent vulvovaginal candidiasis. *Am. J. Obstet. Gynecol.* 214 15–21. 10.1016/j.ajog.2015.06.067 26164695

[B30] Sobel J. D. Sobel R. (2018). Current treatment options for vulvovaginal candidiasis caused by azole-resistant *Candida* species. *Expert Opin. Pharmacother.* 19 971–977. 10.1080/14656566.2018.1476490 29932786

[B31] Spadari C. C. de Bastiani F. Lopes L. B. Ishida K. (2019). Alginate nanoparticles as non-toxic delivery system for miltefosine in the treatment of candidiasis and cryptococcosis. *Int. J. Nanomed.* 14 5187–5199. 10.2147/IJN.S205350 31371955 PMC6636311

[B32] Spadari C. C. Vila T. Rozental S. Ishida K. (2018). Miltefosine has a postantifungal effect and induces *Apoptosis* in cryptococcus yeasts. *Antimicrob. Agents Chemother.* 62:18. 10.1128/AAC.00312-18 29844051 PMC6105859

[B33] Tong Z. Widmer F. Sorrell T. C. Guse Z. Jolliffe K. A. Halliday C. (2007). In vitro activities of miltefosine and two novel antifungal biscationic salts against a panel of 77 dermatophytes. *Antimicrob. Agents Chemother.* 51 2219–2222. 10.1128/AAC.01382-06 17371821 PMC1891392

[B34] Turecka K. Chylewska A. Kawiak A. Waleron K. F. (2018). Antifungal activity and mechanism of action of the Co(III) coordination complexes with diamine chelate ligands against reference and clinical strains of *Candida* spp. *Front. Microbiol.* 9:1594 10.3389/fmicb.2018.01594 PMC6058090 30072969

[B35] Ventin F. Cincura C. Machado P. (2018). Safety and efficacy of miltefosine monotherapy and pentoxifylline associated with pentavalent antimony in treating mucosal leishmaniasis. *Expert Rev. Anti. Infect. Ther.* 16 219–225. 10.1080/14787210.2018.1436967 29411659

[B36] Vila T. Ishida K. Seabra S. H. Rozental S. (2016). Miltefosine inhibits Candida albicans and non-albicans *Candida* spp. biofilms and impairs the dispersion of infectious cells. *Int. J. Antimicrob. Agents* 48 512–520. 10.1016/j.ijantimicag.2016.07.022 27667564

[B37] Vila T. V. Chaturvedi A. K. Rozental S. Lopez-Ribot J. L. (2015). In vitro activity of miltefosine against candida albicans under planktonic and biofilm growth conditions and in vivo efficacy in a murine model of oral candidiasis. *Antimicrob. Agents Chemother.* 59 7611–7620. 10.1128/AAC.01890-15 26416861 PMC4649157

[B38] Vila T. V. Ishida K. de Souza W. Prousis K. Calogeropoulou T. Rozental S. (2013). Effect of alkylphospholipids on *Candida albicans* biofilm formation and maturation. *J. Antimicrob. Chemother.* 68 113–125. 10.1093/jac/dks353 22995097

[B39] Wasunna M. Njenga S. Balasegaram M. Alexander N. Omollo R. Edwards T. (2016). Efficacy and safety of ambisome in combination with sodium stibogluconate or miltefosine and miltefosine monotherapy for african visceral leishmaniasis: phase II randomized trial. *PLoS Negl. Trop. Dis.* 10:e0004880. 10.1371/journal.pntd.0004880 27627654 PMC5023160

[B40] Widmer F. Wright L. C. Obando D. Handke R. Ganendren R. Ellis D. H. (2006). Hexadecylphosphocholine (miltefosine) has broad-spectrum fungicidal activity and is efficacious in a mouse model of cryptococcosis. *Antimicrob. Agents Chemother.* 50 414–421. 10.1128/AAC.50.2.414-421.2006 16436691 PMC1366877

[B41] Wu Y. Totten M. Memon W. Ying C. Zhang S. X. (2020). In vitro antifungal susceptibility of the emerging multidrug-resistant pathogen candida auris to miltefosine alone and in combination with amphotericin B. *Antimicrob. Agents Chemother.* 64:e02063-19. 10.1128/AAC.02063-19 31791945 PMC6985732

[B42] Zuo X. Djordjevic J. T. Bijosono O. J. Desmarini D. Schibeci S. D. Jolliffe K. A. (2011). Miltefosine induces apoptosis-like cell death in yeast via Cox9p in cytochrome c oxidase. *Mol. Pharmacol.* 80 476–485. 10.1124/mol.111.072322 21610197

